# New Saffold Cardioviruses in 3 Children, Canada

**DOI:** 10.3201/eid1405.071675

**Published:** 2008-05

**Authors:** Yacine Abed, Guy Boivin

**Affiliations:** * Centre Hospitalier Universitaire de Québec, Quebec City, Quebec, Canada; † Infectious Disease Research Centre, Quebec City, Quebec, Canada

**Keywords:** Human picornavirus, SAF-V cardiovirus, polyprotein, respiratory infections, children, dispatch

## Abstract

In Canada, cardiovirus isolates related to Saffold virus were detected in nasopharyngeal aspirates from 3 children with respiratory symptoms. Polyprotein sequence of the Can112051-06 isolate had 91.2% aa identity with Saffold virus; however, EF and CD loops of the viral surface varied substantially.

The family *Picornaviridae* contains 9 genera: *Enterovirus, Hepatovirus, Rhinovirus, Kobuvirus,* and *Parechovirus* infect humans, whereas *Aphtovirus, Erbovirus, Teschovirus,* and *Cardiovirus* are animal pathogens (*1*). The genus *Cardiovirus* is divided into 2 species: Theiler viruses and the encephalomyocarditis viruses (EMCVs) (*2*–*5*). Although rats and mice are the natural hosts for EMCVs, these cardioviruses have been found to infect many animal species including pigs, rodents, elephants, macaques, and humans (*6*–*9*). Recently, a new cardiovirus provisionally named Saffold virus (SAF-V) was isolated from a stool sample of an 8-month-old girl with fever (*10*). This virus is believed to constitute a novel cardiovirus species and is more genetically related to Theiler-like virus than to other known cardioviruses (*10*). We report the identification and characterization of 3 SAF-V isolates recovered from children with respiratory symptoms.

## The Patients

**Figure 1:**
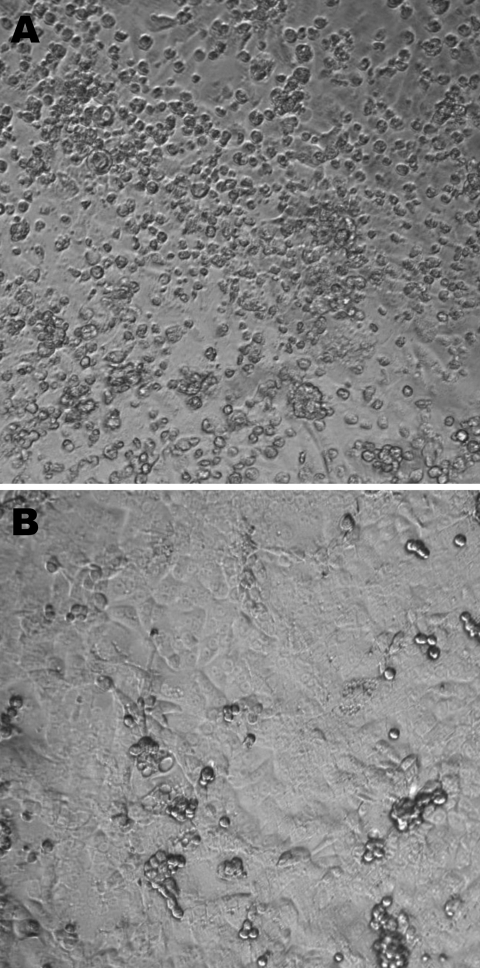
A) Cytopathic effects (round cells) observed 6 days after infection of rhesus monkey kidney (LLC-MK2) cells (second passage) with the Can112051-06 Saffold virus–like cardiovirus strain. B) Uninfected LLC-MK2 cells. Maginification ×10.

The first patient was a 23-month-old girl who was referred on March 6, 2006, to a tertiary hospital for bilateral otitis media that had not responded to amoxicillin or later to cefprozil. She also had cough, rhinorrhea, and fever of 39°C. Her 5-month-old brother had similar clinical signs. Blood cultures were negative, as were antigen detection tests for influenza A and B viruses, the respiratory syncytial virus, and adenoviruses. After 24 hours, the girl was discharged with a diagnosis of bilateral acute otitis media secondary to a viral infection. A nasopharyngeal aspirate collected at the time of admission was inoculated onto different continuous cell lines including human lung adenocarcinoma (A-549); human rhabdosarcoma (RD); transformed human kidney (293); human colon adenocarcinoma (HT-29); human laryngeal carcinoma (Hep-2); human foreskin fibroblast; mink lung; and Vero, MDCK, and rhesus monkey kidney (LLC-MK2) cells. Cultures were incubated for 3 weeks at 37°C in 5% CO_2_. A viral isolate (Can112051-06) with cytopathic effects (round cells) suggestive of a picornavirus was observed only in LLC-MK2 cells after 6 days of incubation (Figure 1). An immunofluorescent assay that used the Pan-Enterovirus Blend kit (Light Diagnostics, Levingston, UK) gave a moderate fluorescent signal. Nucleic acid extracts from Can112051-06 were further analyzed with a multiplex real-time reverse transcription–PCR (RT-PCR) assay for common respiratory viruses (influenza A and B viruses, human respiratory syncytial virus, and human metapneumovirus) (*11*) as well as RT-PCR assays for enteroviruses and parechoviruses (*12*); results were negative.

The supernatant from LLC-MK2–infected cells was treated with DNase and divided into 2 aliquots for DNA and RNA extractions by using the QIAamp Blood Mini Kit and QIAamp Viral RNA extraction kits (QIAGEN, Mississauga, Ontario, Canada), respectively. Nucleic acids were then used in the sequence-independent single-primer amplification method as described (*13*). Amplicons of 800–1,200 bp obtained from RNA samples were cloned and sequenced.

**Figure 2:**
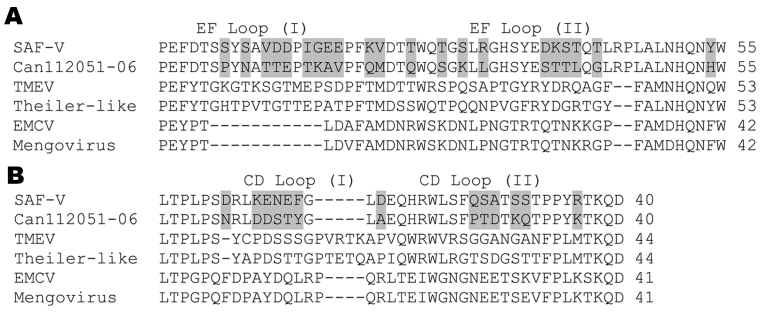
Comparison of amino acid sequences of the A) EF loop structure (part of the VP2 protein) and B) the CD loop structure (part of the VP1 protein) between Can112051-06 and other cardioviruses including Saffold virus (SAF-V), Theiler’s murine encephalomyelitis virus (TMEV), Theiler-like virus, encephalomyocarditis virus (EMCV), and Mengovirus. Amino acid differences between Can112051-06 and SAF-V are shaded.

Sequence determination of cloned amplicons followed by tBLASTx analysis showed similarity of Can112051-06 sequences with the SAF-V VP4 and 2C sequences (data not shown). Subsequent PCR amplifications and sequencing reactions that used primers selected from our clones and the complete SAF-V genome sequence (GenBank accession no. EF165067) enabled us to determine the complete polyprotein encoding region of the Can112051-06 isolate (GenBank accession no. AM922293). This region was 6,879 nt long compared with 6,888 nt for the SAF-V polyprotein sequence; nucleotide identity between the 2 strains was 82.5%. The Can112051-06 polyprotein comprised 2,293 aa compared with 2,296 aa for the SAF-V polyprotein; amino acid identity between the 2 strains was 91.2%. Deletions of 1 aa in the VP2 and 2 in the VP1 proteins were found in Can112051-06 with regard to the prototype SAF-V strain. As expected, the Can112051-06 and SAF-V polyproteins contained 11 putative cleavage sites. The 8 aa flanking these sites were conserved; 6 sites were identical in the 2 strains, whereas the remaining sites had 1- or 2-aa differences (Table 1). The resulting 12 proteins of Can112051-06 and SAF-V had 76.1%–100% aa identities (Table 2). The highest difference level was seen in the L peptide. In addition to the L peptide, some cardioviruses, in particular Theiler’s murine encephalomyelitis virus strains that are associated with persistent infections, contain an alternate open reading frame (ORF), the so-called L* (*14*). As for the prototype SAF-V strain, the Can112051-06 putative L* ORF is unlikely to encode a protein because it has an ACG (instead of ATG) start codon (data not shown). In addition, contrasting with the SAF-V L*, which contained 57 aa, the Can112051-06 L* sequence contained only 34 aa. Comparison of the L* sequence of Can112051-06 with the first 34 aa of the SAF-V L* sequence showed 60.6% identity (data not shown). Four small loops are exposed on the virion surface of cardioviruses; 2 are part of the VP2 EF loop structure, and 2 are part of the VP1 CD loop structure. The EF loop structure of Can112051-06, which contained 55 aa (residues 274-328 of the polyprotein), had 61.8% aa identity with that of SAF-V (Figure 2, **panel A**). Similarly, the CD loop structure of Can112051-06, which contained 40 aa (residues 712-751 of the polyprotein), had 67.5% aa identity with the SAF-V counterpart (Figure 2, **panel B**).

**Table 1:** Cleavage sites of Can112051-06 and prototype Saffold virus cardiovirus polyproteins*

Cleavage site	Can112051-06	Saffold virus
L / VP4	MEPQ / GNSN	MEPQ / GNSN
VP4 / VP2	PLLM / DQNT	PLLM / DQNT
VP2 / VP3	LEDQ / SPIP	LEAD / SPIP
VP3 / VP1	YTPH / GVDN	YTPQ / GVDN
VP1 / V2A	LELQ / NPIS	LELQ / DPIS
2A / 2B	FQLQ / GGVL	FQLQ / GGVL
2B / 2C	LQQQ / SPVR	LQQQ / SPIR
2C / 3A	LVAQ / SPGN	LVAQ / SPGN
3A / 3B	EGEQ / AAYS	EGEQ / AAYS
3B / 3C	LDVQ / GGGK	LDVQ / GGGK
3C / 3D	LIPQ / GAIV	LTPQ / GAIV

*Can112051-06 GenBank accession no. AM922293; Saffold virus GenBank accession no. EF165067.

**Table 2:** Amino acid identities between Can112051-06 and prototype Saffold virus proteins*

Protein	% Identity
L	76.1
VP4	97.2
VP2	83.9
VP3	85.2
VP1	76.7
2A	95.8
2B	97.6
2C	96.6
3A	100
3B	95.0
3C	96.8
3D	97.0

*Can112051-06 GenBank accession no. AM922293; Saffold virus GenBank accession no. EF165067.

Other respiratory samples with picornavirus-like cytopathic effects on LLC-MK2 cells and weakly immunofluorescent signals according to the Pan-Enterovirus Blend Kit were screened for cardiovirus SAF-V by using a specific RT-PCR assay targeting a 2A–2C encoding region (1,407 nt, 469 aa). With use of this strategy, 2 more cases were noted in September 2006: 1 in a 19-month-old child hospitalized for suspected bacteremia and a cold and 1 in a 4-year-old child hospitalized for right lung pneumonia and otitis media. The 2A-2C aa sequences of these additional isolates were identical and shared 96.6% and 97.2% aa identities with the corresponding regions of Can112051-06 and the prototype SAF-V, respectively.

## Conclusions

Our findings suggest a pathogenic role for SAF-V-like viruses in humans. Although the polyprotein sequences of the Can112051-06 strain and the original US strain were related, the EF and CD loop structures varied substantially (61.8% and 67.5% aa identities, respectively). For comparison, the EF and CD loop structure sequences of EMCV and Mengovirus (2 members of the EMCV species) have 95.2% and 95.1% aa identities, respectively. The difference between time of isolation of SAF-V (1981) and the Can112051-06 strain (2006) is unlikely to be responsible for such a high level of sequence variation. We previously showed that the amino acid sequences of the VP0-VP1 capsid region of Canadian human parechovirus 1 strains isolated from 1985 through 2004 had 89.2% to 97.5% identities (*12*). Because the EF and CD loop structures are exposed on the viral surface of cardioviruses and thus constitute an important site for recognition by neutralizing antibodies (*15*), Can112051-06 and the original SAF-V might represent different serotypes, although further serologic studies are needed to confirm this hypothesis. The implication of the weak immunofluorescent signal seen in cardiovirus-infected cells stained with an enterovirus antibody is uncertain because of the considerable difference between the capsid proteins of cardioviruses and enteroviruses, which constitute 2 separate picornavirus genera.

In contrast to the initial recovery of this virus from a stool sample (*10*), our 3 strains were recovered from nasopharyngeal aspirate samples of children with fever and some other respiratory signs. The cardioviruses were the only pathogens identified in these samples. Whether SAF-V and the related Canadian strains described in this study should be classified as a new human *Cardiovirus* species or as a new clade within the *Theilovirus* species remains to be determined.
